# Automated Counting Grains on the Rice Panicle Based on Deep Learning Method

**DOI:** 10.3390/s21010281

**Published:** 2021-01-04

**Authors:** Ruoling Deng, Ming Tao, Xunan Huang, Kemoh Bangura, Qian Jiang, Yu Jiang, Long Qi

**Affiliations:** 1 College of Engineering, South China Agricultural University, Guangzhou 510642, China; drl1207@stu.scau.edu.cn (R.D.); taoming@stu.scau.edu.cn (M.T.); huangxunan@stu.scau.edu.cn (X.H.); k.bangura@slari.gov.sl (K.B.); jiangqian@stu.scau.edu.cn (Q.J.); 2 Modern Educational Technology Center, South China Agricultural University, Guangzhou 510642, China; nova_yy@scau.edu.cn; 3 Lingnan Guangdong Laboratory of Modern Agriculture, Guangzhou 510642, China

**Keywords:** grain detection, primary branch, convolutional neural network, image, rice

## Abstract

Grain number per rice panicle, which directly determines grain yield, is an important agronomic trait for rice breeding and yield-related research. However, manually counting grains of rice per panicle is time-consuming, laborious, and error-prone. In this research, a grain detection model was proposed to automatically recognize and count grains on primary branches of a rice panicle. The model used image analysis based on deep learning convolutional neural network (CNN), by integrating the feature pyramid network (FPN) into the faster R-CNN network. The performance of the grain detection model was compared to that of the original faster R-CNN model and the SSD model, and it was found that the grain detection model was more reliable and accurate. The accuracy of the grain detection model was not affected by the lighting condition in which images of rice primary branches were taken. The model worked well for all rice branches with various numbers of grains. Through applying the grain detection model to images of fresh and dry branches, it was found that the model performance was not affected by the grain moisture conditions. The overall accuracy of the grain detection model was 99.4%. Results demonstrated that the model was accurate, reliable, and suitable for detecting grains of rice panicles with various conditions.

## 1. Introduction

Rice (*Oryza sativa*), as a significant food crop, is widely cultivated all over the world. The grain counts per panicle at the mature stage are critical data for rice breeding research and yield assessment [1,2]. Additionally, it is viewed as one of the key traits for genetic improvement of rice yield [3,4,5]. Therefore, detection of the number of grains per panicle of rice is of great importance.

The traditional method of counting grains from a panicle is to thresh the rice panicle first and then manually count the grains. This method has several shortcomings. For example, some grains may get lost during the threshing process, some grains may get mechanically damaged, and some other grains may be still attached to the rice panicle. These shortcomings may lead to some errors. Due to the error of the original method and the error caused by the lost grains, the final cumulative error of the traditional method will increase. This error cannot be ignored for rice yield evaluation and breeding. Because breeding requires precise knowledge of the total grain number per rice panicle. Also, in the process of yield estimation, there are about one million stubbles of rice per acre, and each stubble has about nineteen effective panicles. If the number of grains per panicle is wrong, it will seriously affect the final yield estimation. On the other hand, manually counting a large number of grains is inefficient. Therefore, it is necessary to develop an efficient technology to automatically detect and count grains. 

Existing technologies have been focused on counting grains using traditional image processing methods. For example, visible light imaging and soft X-ray imaging methods were combined to count grains on panicle [6]. Others involve an engineering prototype combining traditional image processing methods such as image segmentation and median filtering [7], and a P-TRAP software for this purpose [8]. Attention has been paid to the development of grain counting algorithms based on other analyses, such as a Fourier analysis [9], corner detection and neural network classification [10], as well as contour curvature analysis [11]. Although these image-based methods were proven viable for counting grains with reasonable accuracies, their application was to count grains after threshing, which caused errors as afore-mentioned. Direct counting the grains on the panicle before threshing can avoid those errors. However, little research has been done in this regard.

Few methods have been explored to count grains directly on the panicle. For example, a panicle-image based method integrating image analysis and a five-point calibration model was proposed for spikelet number per panicle (also known as grain number per panicle) [12]. An image-based prior edge wavelet correction model was also developed for grain counting on rice panicles [13]. Although these methods were workable for obtaining grain number per panicle, their accuracy still needs to be improved. Deep learning, which is a novel method for object detection with greater accuracy, has been widely used for agricultural applications [14]. These include the detection and counting of corn kernels [15], the leaf counting in maize plants [16], the detection and analysis of wheat spikes [17], seed-per-pod estimation for plant breeding [18], and automatic estimation of heading date of rice [19]. Deep-learning based image analysis has broad prospects and can be used to accurately and effectively detect and count grains per panicle. 

A deep learning model for automatically counting grains on the rice panicle by [20] had higher counting accuracy. However, this method failed to identify partially covered grains due to the few pixels of small objects. There is a need for improving the deep learning method so that it can successfully detect small grains. The objectives of this study were to: (a) develop a model to automatically recognize grains on rice panicles based on CNN deep learning, (b) evaluate the accuracy of the grain detection model using images taken from rice fields, and (c) verify whether the model can be applied to different grain moisture conditions.

## 2. Materials and Methods

### 2.1. Image Collections

#### 2.1.1. Description of Image Capture

**Figure 1:**
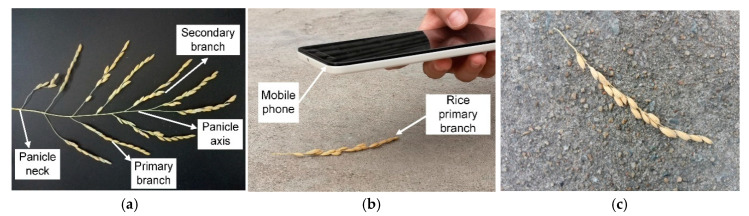
Illustration of the process of image capturing: (**a**) structure of a rice panicle; (**b**) rice primary branch and image taking with a mobile phone; (**c**) image captured showing the rice primary branch.

A rice panicle has a complex branching structure consisting of a main axis, a neck, and lateral branches (Figure 1a). These lateral branches are called primary branches from which secondary branches extend. Third branches are not common and may be observed from some hybrid rice. Grains located in a whole rice panicle are very small objects in an image, and the overlapping grains will be even smaller. Such a smaller grain has too few pixels to provide enough information for detection. It would be difficult to perfectly recognize every grain through a whole panicle image. Therefore, primary branch images were collected in this study. 

Images were taken from a paddy field during the rice maturity state. The field was located at the Institute of Agricultural Sciences in Jiangmen, Guangdong province, China (22°34′49.404″ N, 113°4′48.036″ E). Firstly, a fresh primary branch was detached from the panicle and was placed on the ground (Figure 1b). Then, an RGB image of the primary branch was taken at 60–110 mm above the ground from the nadir direction using a mobile phone with a camera resolution of 3968 × 2976 pixels. A primary branch of rice panicle captured in the image is shown in Figure 1c. Separate sets of images were collected for model development and model verification. Details on rice variety and environmental condition are described in the following section.

#### 2.1.2. Image Sets

One set of rice primary branch images (referred to as Original image set hereafter) was collected to establish the grain detection model. Images were taken in the paddy field on 1–3 July 2019 when the rice was mature, under different environmental conditions. The rice variety was Guguangyouzhan belonging to Indica rice. The grain morphology of this rice variety was slightly thin and long. In total, 796 images were collected. Among them, the number of images taken was 378, 315, and 103 for sunny, cloudy, and blurred conditions, respectively.

The other set of rice primary branch images (referred to as Verification image set hereafter) was collected to verify if the grain detection model could be robustly applied to different grain moisture conditions. Images were taken on 1 July 2020 at the rice maturity stage in the same field, but with different rice variety, Zhenguiai that has relatively fatter and shorter grains. In the rice yield measurement, the manual grain count is usually directly performed on the newly harvested rice panicle, while in the rice variety improvement study, the grain on the rice panicle is counted after drying. Through observation, it was found that, due to the scattered growth of the branches directly connected to the grains, the degree of occlusions between the grains on the branches of the newly harvested rice panicle was slightly less. After drying, due to lack of water, the small branches on the rice panicle branches will slightly shrink and gather in the direction of the main branches, which will increase the overlap between the grains. Therefore, it was expected that grain moisture conditions may have affected on grain detection results, as different grain moistures may have different occlusions on images. Images were taken using the same mobile phone at two moisture conditions: fresh and dry. The difference between fresh and dry is that the color of freshly harvested grains is bright yellow, and the yellow will fade slightly after drying. First, 35 images of mature fresh rice primary branches were taken, then the 35 branches were exposed to the sun to let them completely dry, and the images of the dried branches were taken. In summary, for each grain moisture condition, 35 images were collected, totaling to 70 images. The detailed information of the two image sets can be seen in Table 1.

### 2.2. Grain Detection Method

The faster R-CNN [21] model based on feature pyramid networks (FPN) [22], which is effective for multi-scale object detections, was used for grain detection. The grain detection model based on Faster R-CNN with FPN was trained using the images of the Original set. For this, the images needed to be preprocessed, as discussed in the following sections.

#### 2.2.1. Image Annotation

**Figure 2:**
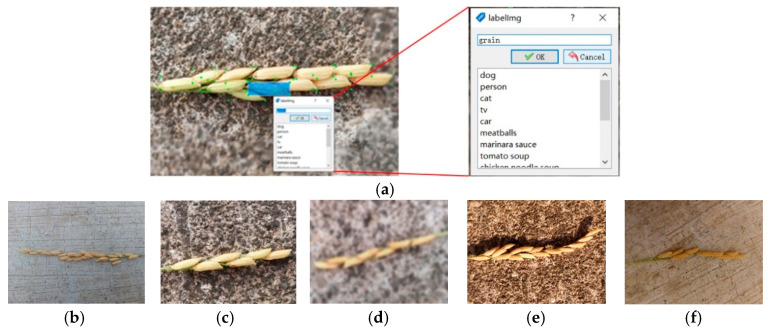
Examples of labeling grains with bounding boxes: (**a**) drawing bounding boxes using the LABELIMG software; (**b**) small scale, (**c**) large scale, (**d**) blurred condition, (**e**) sunny condition with background shadows, (**f**) cloudy condition.

To maintain the data consistency and reduce computing memory, the longest side of the images in the Original image set was uniformly scaled to 1280 pixels, and the shortest side was scaled accordingly to the image aspect ratio. The image annotation process was completed using the LabelImg annotation tool [23]. The annotation process had mainly two steps: drawing a rectangular frame around a grain (Figure 2a), and storing the labels and coordinates of the rectangular frame in the XML file, in the same format of PASCAL VOC dataset used by ImageNet [24]. Finally, each image in the dataset had a corresponding XML format annotation file.

When the image was collected, the lighting condition was different. Besides, during image acquisition, the accidental shaking of hand may result in blur occurrence. As the results, the appearance of grains had different scales and clarities in the images, including small scales (Figure 2b) or large scales (Figure 2c), blurred conditions (Figure 2d), and sunny (Figure 2e) or cloudy environments (Figure 2f). To increase the robustness of the model, these images were carefully labelled. Also, when the occluded area of grain was more than 90% or when the area of the grain located on the edge of the image was less than 10%, this grain was not labelled. After the annotation process was done, the 796 images in the Original image data set were randomly separated into training, validation, and testing sub-sets with the ratio to the total images of 0.56, 0.24, and 0.2, respectively.

#### 2.2.2. Grain Detection Based on Faster R-CNN with FPN

**Figure 3:**
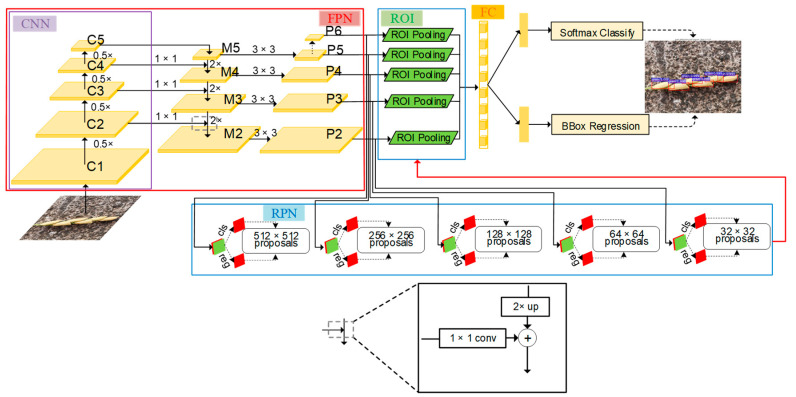
The architecture of the grain detection model.

Figure 3 shows a schematic diagram of the Faster R-CNN with FPN network used in this study. Faster R-CNN with FPN was comprised of three parts: FPN for generating multi-scale feature maps, a region proposal network (RPN) using these multi-scale feature maps for generating multi-scale region proposals for objects, and a Fast R-CNN using these multi-scale proposals to detect objects. The backbone CNN extracted multi-scale feature maps of the original images through a set of basic conv+relu+pooling layers. The FPN network uses the inherent multi-scale pyramid structure of the deep convolutional neural network to construct a feature pyramid. Specifically, this is to up-sample the feature map of the highest layer of the convolutional neural network (i.e., 2× large the size) and then add it to the feature map of the lower layer of the convolutional network after 1 × 1 convolution (horizontal connection) to form a layer of the M feature layers. Follow this operation, each layer of the M feature layers was built from top to bottom layer by layer. After each feature layer in the M feature layers undergoes 3 × 3 convolution, the feature pyramid was obtained. The RPN was used to generate multi-scale region proposals through multi-scale feature maps produced by FPN. Both of the multi-scale feature maps and region proposals were fed into an ROI (Region of Interest) pooling layer to obtain the proposal feature maps. The prediction of the grain is carried out through feeding the proposal feature maps into the fully connected layer.

FPN consists of two parts: the first part is the process of bottom-up, and the second part is the fusion process of top-down and lateral connection. 

In the bottom-up process, CNN networks are divided into different stages according to the size of the feature map, and the scale ratio of the feature map between each stage differs by two. Among them, each stage corresponds to a feature pyramid level, and the last layer of each stage feature is selected as the feature corresponding to the corresponding level in FPN. Taking ResNet as example, the last residual block layer features of conv2, conv3, conv4, and conv5 layers are selected as the features of FPN, which are recorded as {C2, C3, C4, C5}. The steps of these feature layers relative to the original image are 4, 8, 16, and 32, respectively. 

The top-down process uses up-sampling to enlarge the small feature map on the top layer (such as 20) to the same size as the feature map of the previous stage (such as 40). The advantage of this is that it not only utilizes the strong semantic features of the top layer (facilities classification), but also uses the high-resolution information of the bottom layer (facilitates positioning). The up-sampling method can be implemented with the nearest neighbor difference value. In order to combine the high-level semantic features with the bottom-level precise positioning capabilities, a lateral connection structure similar with the residual network is used. The lateral connection merges the features of the upper layer that have the same resolution as the current layer after up-sampling through the addition method. (Here, in order to correct the number of channels, the current layer is subjected to a 1 × 1 convolution operation.) The specific schematic diagram can be seen in the FPN part of Figure 3.

Specifically, the C5 layer first undergoes 1 × 1 convolution to obtain M5 features. M4 layer was obtained by up-sampling the M5 and then plus the C4 layer after 1 × 1 convolution. Do this process two more times to get M3 and M2, respectively. The M layer features are then subjected to 3 × 3 convolution to obtain the final P2, P3, P4, and P5 layer features. Since each P layer has different scale information relative to the original image, the scale information in the original image was separated to make each P layer process only a single scale information. Specifically, the anchor of the five scales {32^2^, 64^2^, 128^2^, 256^2^, 512^2^} correspond to the five features {P2, P3, P4, P5, P6}. Each feature layer processes three candidate frames with 1:1, 1:2, and 2:1 aspect ratio. P6 is specifically designed for RPN networks and was used to process 512-dimensional candidate boxes. It is obtained by down-sampling from P5.

Each feature layer of the FPN was compared to the features of each level of the image pyramid, thereby the regions of interest (ROI) were mapped to the corresponding feature layers. Taking the input of 224 size pictures as an example, the ROI with width and height will be mapped to the feature level k, and its calculation formula is as follows:

(1) $$k=\left\lfloor k_{0}+\log_{2}\left(\sqrt{wh}/224\right)\right\rfloor$$

where *k* is the feature level, *k*_0_ is 4, *w* is the width of the ROI, and *h* is the length of the ROI.

In ResNet, the value of *k*_0_ is 4, which corresponds to the level of the box with a length and width of 224. If the length and width of the box are divided by 2 related to 224, then the value of *k* will be reduced by 1, and so on.

#### 2.2.3. Training of the Grain Detection Model

The training and verification image sub-sets separated in Section 2.2.1 were served as inputs for transferring learning using the pretrained ResNet 50 network. The algorithm was implemented based on the deep learning framework Pytorch written in Python, primarily developed by Facebook’s AI Research lab (FAIR) and executed on a graphics workstation. Detailed information of the hardware and software was provided in Table 2. The training processes for the model were done under the conditions with epoch of 228, learning rate of 0.001, momentum of 0.9, and weight decay of 0.0001. When the loss function converged and stabilized, training was stopped, and the training model was saved.

### 2.3. Evaluation Metrics

To verify the generalization ability and accuracy of the trained model, the precision rate, recall rate, as well as the accuracy of the model were evaluated. Also, the intersection over union (IOU) [25], based on Jaccard index, was used to evaluate the overlap between labelled bounding box and detected bounding box. The standard IOU threshold value of 0.9 was used. The IOU is defined in Equation (2).

(2) $$IOU\left(B_{p},B_{l}\right)=\frac{area\left(B_{p}\cap B_{l}\right)}{area\left(B_{p}\cup B_{l}\right)}$$

where *B_p_* and *B_l_* are the predicted bounding box and the labelled bounding box, respectively; *B_p_* ∩ *B_l_* is the intersection of the detected bounding box and the ground truth bounding box. *B_p_* ∪ *B_l_* is the union of two boxes.

If grain was surrounded by a detected bounding box, the detected bounding box was regarded as correctly detected (true positive, TP). Inversely, if the background was surrounded by a detected bounding box, the detected bounding box was regarded as mistakenly detected (false positive, FP). When grain could not be detected, it was regarded as false negative (FN). The precision and recall were then calculated by Equations (3) and (4).

(3) $$Precision=\frac{TP}{FP+TP}$$

(4) $$Recall=\frac{TP}{FN+TP}$$

(5) $$Accuracy=\frac{TP+TN}{TP+TN+FP+FN}$$

where *P* is precision; *R* is recall; *TP* is the total number of correctly detected grains; *FP* is the total number of incorrectly detecting background regions as grains; *FN* is the total number of incorrectly detecting grains as background regions; *TN* is the correct identification of background which is always ‘zero’ and is not needed to be used in a binary classification problem that always determines the foreground for object detection; *FP* + *TP* represents for the total numbers of detected grains; *FN* + *TP* represents for the total numbers of true grains. The accuracy curve in Equation (5) was plotted to evaluate the detection performance of the grain model.

Finally, to further verify the applied robustness of the grain detection model, the Verification image set of 70 images (35 for fresh and 35 for dried rice primary branches) were used to test. Then, the testing results were compared with manual counting results. Several metrics were used to evaluate the agreement between the two sets of results: the coefficient of determination (*R*^2^), root mean square error (RMSE), the relative RMSE (rRMSE), and mean absolute error (MAE). These metrics were computed using the following equations:

(6) $$R^{2}=1-\frac{\sum_{j=1}^{n}{\left(m_{j}-e_{j}\right)}^{2}}{\sum_{j=1}^{n}{\left(m_{j}-\bar{m}\right)}^{2}}$$

(7) $$RMSE=\sqrt{\frac{\sum_{j=1}^{n}{\left(e_{j}-m_{j}\right)}^{2}}{n}}$$

(8) $$rRMSE=\sqrt{\frac{1}{n}{\sum_{j=1}^{n}\left(\frac{m_{j}-e_{j}}{m_{j}}\right)}^{2}}$$

(9) $$MAE=\frac{\sum_{j=1}^{n}\left|e_{j}-m_{j}\right|}{n}$$

where *m_j_* and *e_j_* are the manual calculation and model detection of image *j*, respectively; *n* is the total number of the detected images.

## 3. Results and Discussion

### 3.1. The Behavior of the Grain Detection Model during the Training Process

**Figure 4:**
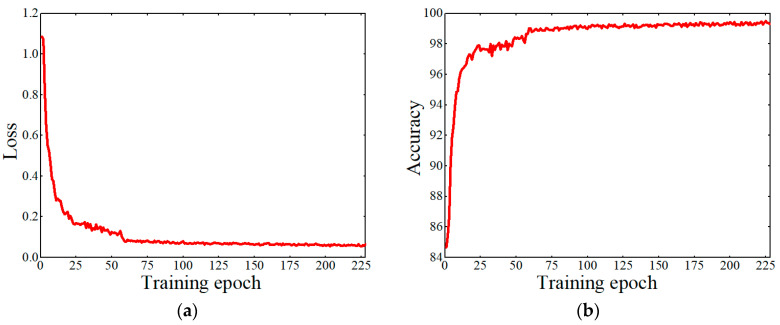
The changes of loss and accuracy values during training process of the grain detection model: (**a**) loss; (**b**) accuracy.

Figure 4 showed how the model loss and accuracy changed during the training process. The loss value gradually decreased with the increase in training epochs (Figure 4a). The loss value of the first 25 training epochs changed rapidly. Afterward, the change was slow down. After 60 epochs, the loss value remained fairly stable in the range from 0.05 to 0.06. Hence, the training was suspended at 228 epochs. While the loss was decreasing, the accuracy value was increased with the increase in training epochs (Figure 4b). The accuracy of the model experienced a rapid increase during the first 25 training epochs, and a slowing down from 25 to 60 epochs. After 60 epochs, the accuracy value tended to be stable. The loss and accuracy values fluctuated slightly in different epochs, but the main trends eventually converged. Therefore, the training was successfully completed.

### 3.2. Performance of the Grain Detection Model

Next, the model was used for grain detection using the 160 testing sub-set images from the Original image set. The detection results are shown in Table 3 for confidence values from 0.4 to 0.9. They were compared with the manual counting results of 1779 grains. Over all cases, more than 1770 grains were correctly detected (true positive). The number of incorrected identifications (false positive) were found to be from 0 to 17, and only few grains were missed. The resultant precision rate was as high as 100%. Even at the confidence value of 0.4, the precision rate could still reach 99.0%. The recall rate was 99.5% and above, over the confidence value from 0.4 to 0.9. Over the range of confidence value, the mean accuracy was found to be from 98.8 to 99.7%, with an average of 99.4%. This proved that the performance of the grain detection model was good and stable. 

### 3.3. Comparison with the Other C-NN Models

**Figure 5:**
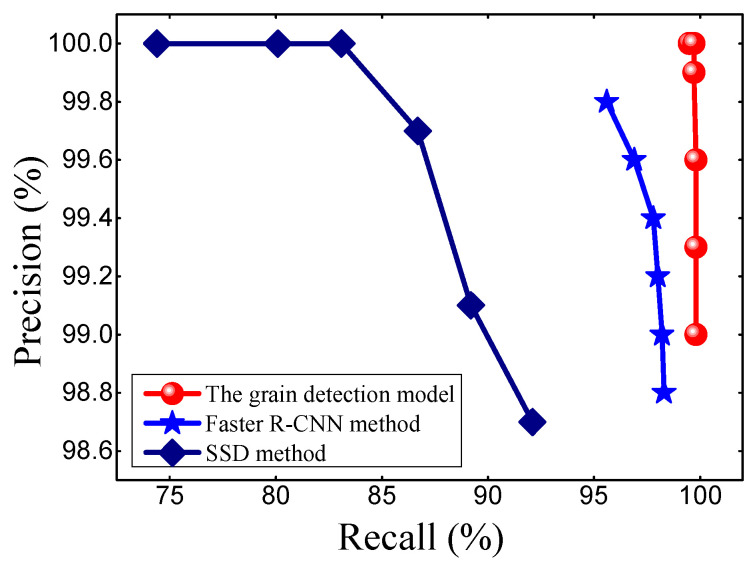
The Precision-Recall curve of the grain detection model compared with the original Faster R-CNN method and SSD method.

To further verify the grain detection model (faster RCNN combined with FPN), the model performance was compared with that of the original faster R-CNN model and the SSD model. The same data set was used for training the faster R-CNN model and SSD model, respectively. The P-R curves for the three models during testing are shown in Figure 5. The area under the P-R curve of the grain detection model was larger than that of the Faster R-CNN method and the SSD method, which demonstrated that the grain detection model had better performance in detecting grains. The main reason for the better performance was that the grain detection model had a better capability in detecting different scales of grains.

Moreover, the three models were compared in detail, choosing 0.9 as the critical confidence value. Table 4 showed that under the same confidence value, the grain detection model could correctly detect 1770 grains out of 1779 which were identified manually, the number of false detections was zero, and the number of missed detections was only 9. The corresponding results of the original faster R-CNN model were 1707, 3, and 72. Also, the corresponding results of the SSD model were 1324, 0, and 455. As the result, the precision rate, recall rate, and accuracy of the grain detection model all were higher than those of the original faster R-CNN model. Further, the recall and accuracy of the grain detection model were much higher than those of the SSD model, although the precision of the grain detection model was equal to that of the SSD model. Therefore, it can be concluded that the grain detection model performed better than the faster R-CNN model. These further demonstrated that the grain detection model (faster RCNN with FPN) had improved performance.

**Figure 6:**
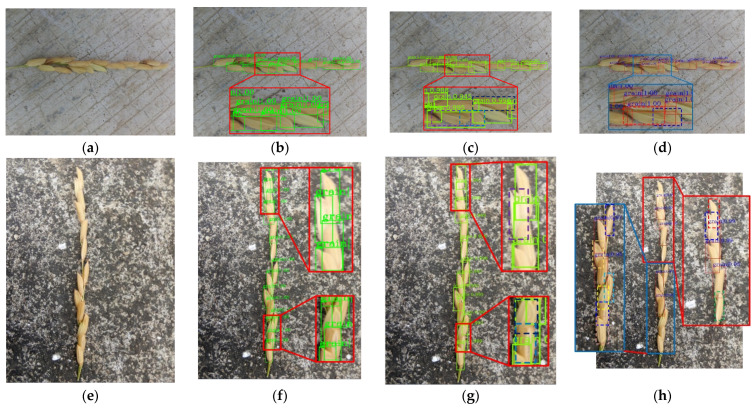
Three examples that show the grain detection results using the grain detection model, the original Faster R-CNN model and SSD model with a cutoff confidence value of 0.9; the texts indicate the confidence scores; (**a**,**e**): original image; (**b**,**f**): detection results of grain detection model; (**c**,**g**) detection results of the Faster R-CNN model; (**d**,**h**) detection results of the SSD model.

Examples of grains detected by the three models and their corresponding confidence values were displayed in Figure 6. When the grain color was similar with the background color, the faster R-CNN model and SSD model usually incorrectly recognize these grains as the background, whereas the grain detection model could completely avoid these errors. When more than half of the area of grains was covered, the Faster R-CNN model would miss these grains, but the grain detection model can perfectly detect these grains. This means that the incomplete information of grain did not affect the detection accuracy of the grain detection model. When the grains are relatively small and the color is brighter, the SSD model will incorrectly recognize these grains as the background, but the grain detection model can be completely unaffected and can perfectly recognize these grains. Also, detection bounding boxes of the grain detection model were more perfect in enclosing the grains, when compared to those of the faster R-CNN model and SSD model. Therefore, the score of the grain detected by the grain detection model was usually higher than that of the faster R-CNN model and SSD model. The high scores (up to 1.000) demonstrated that the grain detection model was quite reliable.

**Figure 7:**
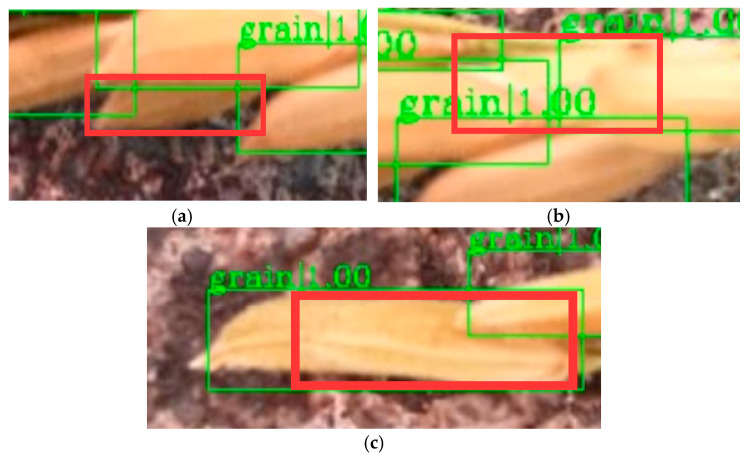
Examples of images causing grain detection errors: (**a**) most area of the grain was covered and blurred; (**b**) most area of the grain was covered and under lighting reflection; (**c**) two bonded grains under light reflection.

After analyzing the false positive and false negative cases, it was found that the subtle discrepancy between the model results and the manual counting results can be attributed to the following reasons. When most area of grain was obscured by other grains and in a dark and fuzzy condition, the grain was easily mistakenly identified as the background (Figure 7a). When the surrounding area of the grain was covered by other grains and under the condition of blurred reflection, the grain was easy to be missed (Figure 7b). These two situations led to the emergence of false negative cases, and accounted for 30% of the nine false negative cases. Also, when two grains overlapped in the same direction and under a light reflect condition, the two grains sometimes could be mistaken as one grain (Figure 7c), which reduced the recognition accuracy. The other 70% of the nine false negative cases were caused by this situation. However, in general, all these situations are rare and can be avoided in the process of taking images.

### 3.4. Further Analysis of Testing Results

The main purpose of this research was to provide researcher and breeders with a fast and accurate algorithm for counting grain number per rice panicle. The algorithm should be able to perform the task under various shooting conditions such as lighting condition and the number of grains on the branch. Therefore, further statistical analysis was carried out to verify the accuracy of the proposed method under these different conditions. 

#### 3.4.1. Effects of the Number of Grains

It was expected that the grain number per primary branch may have effects on grain detection results, as the images with different numbers of grains had different receptive fields. If there was only one grain in an image, the grain would be usually complete, clear, and highly recognizable. However, in reality, an image contains multiple grains, these grains would be minimally identified in the cases where two or more clusters of grains were occluded. Generally, there were 6–9 grains on each primary branch, and 3–5 grains on each secondary branch. Therefore, 160 images in the test set were grouped into three categories, namely 1–9, 10–14, and greater than 14, to see if the number of grains had effects on the grain detection accuracy. 

Results showed that, with an increase in the number of grains in the image, the recall rate had a slight downward trend because more grain overlaps and occlusion appeared in the image (Table 5). However, the precision rate and recall rate didn’t change much, and both were above 99.0%. This was attributable to the good performance of the proposed algorithm in dealing with grains of different scales. Few false negatives occurred where images had larger number of grains, causing overlap and occlusion of grains. Also, the shooting distance of an image with large number of grains was usually longer, and the grains in the image were small, which negatively affected the grain detection, resulting in false recognitions.

#### 3.4.2. Effects of Lighting Conditions

Different lighting conditions may also affect the detection accuracy, since images taken under sunny condition were usually bright and clear, while images taken under cloudy condition were darker and blurry. The 160 images in the test set were divided into two groups according to the lighting conditions: sunny and cloudy. The precision rate of the detection results of the images taken under two lighting conditions remained unchanged, while the recall rate under cloudy condition was slightly lower than that of sunny condition (Table 6). However, in general, the detection accuracy was very high, which indicated that lighting condition had little effect on the detection performance of the model.

### 3.5. Application of the Grain Detection Model

The grain detection model was applied to another rice variety and two different grain moisture conditions (fresh and dry), and the detection results shown in Table 7. Out of 446 grains, 444 grains were correctly identified, two grains were missed, and no grains were mistakenly identified for the fresh primary branches. While for the dry primary branches, 443 grains were correctly identified, three grains were missed, and two grains were incorrectly identified. The detection precision rate and recall rate of the fresh primary branch were slightly higher than those of the dry primary branch. The reason for the slight difference can be summarized as follows. Grains on the fresh primary branches were usually more scattered, and the color of the grains was brighter, which were easier to detect. The grains on the dry primary branches had shrunk due to the shrinkage of the branches. The grains on the dry branches also had more dragons, and the color was relatively tarnished, which would increase the difficulties for grain detection. However, the precision and recall values were similar between the two grain moisture conditions, and both were over 99% in all cases, indicating that the performance of the grain detection model was reliable and not affected by the grain moisture condition.

**Figure 8:**
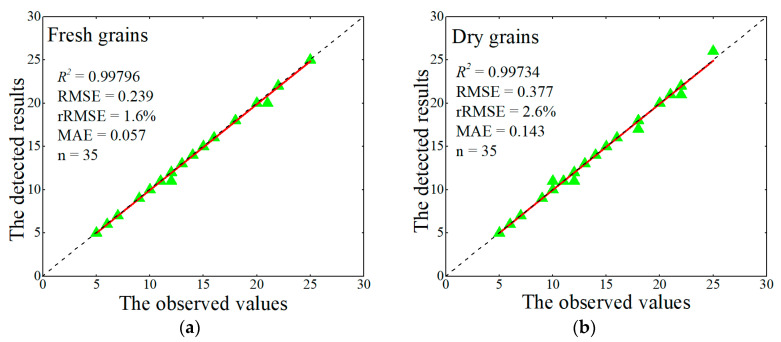
Comparison between the manual observation and model results for counting grains. The solid red line is regression line, the black dashed line is 1:1 line. (**a**) fresh grains; (**b**) dry grains.

To further examine the accuracy of the grain detection model, model results were compared with the manually observed values. Figure 8a showed that fresh grains estimated by the proposed model had relatively good agreement with the observed values, with extremely low errors and a high coefficient of determination (*R*^2^ = 0.998). Besides, the regression line was highly consistent with the 1:1 line. Also, the model had similar results for detecting dry grains (Figure 8b). These demonstrated that the proposed grain detection model can be applied to another rice variety, and provide accurate grain detections, regardless of the grain moisture condition.

## 4. Conclusions

In this study, a high-precision grain detection model was established and tested based on the deep learning convolutional neural network (CNN) for automatic detection and counting of grains per rice panicle. The following conclusions were drawn. The grain detection model, based on faster R-CNN with feature pyramid network (FPN), was capable of detecting grains on the rice panicles under different conditions. The model proposed was found to perform better for recognizing and counting grains per rice primary branch compared to faster R-CNN method along, in terms of precision, recall rate, and accuracy. The mean accuracy of the model was found to be reaching 99.4%, when compared to the results from manual counting of grains. Also, the model could be applied to different numbers of grains per primary branch and various lighting conditions. The detection performance was not affected by the rice varieties and grain moisture conditions. A further step should involve implementing the grain detection model in a smart-phone based APP for convenient and easy applications in the daily life. In addition, the model, after some modifications, can be potentially applied to other crops, such as wheat and corn.

## Figures and Tables

**Table 1:** Detailed information of image sets.

Image Set	Rice Variety	No. of Samples	Imaging Conditions	No. of Samples
Original image set	Guguangyouzhan	796	sunny	378
			cloudy	315
			blurred	103
Verification image set	Zhenguiai	70	fresh	35
			dry	35

**Table 2:** The hardware and software configurations for the deep learning model.

Project	Content
CPU	Intel I7-9700k@3.6GHz x8
RAM	62G
GPU	GeForce GTX 1080 Ti
GPU memory	11G
Operating System	Ubuntu 16.04 LTS
Cuda	Cuda 9.0 with Cudnn v7
Data processing	Python 3.6, OpenCV, LabelImg, etc.
Deep learning framework	Pytorch
Deep learning algorithm	Faster RCNN ResNet50 with FPN

**Table 3:** Precision and recall from grain detection model using testing set images at different confidence values (0.4, 0.5, 0.6, 0.7, 0.8, 0.9) set as the cutoff points.

Confidence Value	Manual Grain Counting	Correctly Identified (True Positive)	Incorrectly Identified (False Positive)	Missed Grain (False Negative)	Precision (%)	Recall (%)	Accuracy (%)
0.9	1779	1770	0	9	100.0	99.5	99.5
0.8	1779	1774	0	5	100.0	99.7	99.7
0.7	1779	1774	1	5	99.9	99.7	99.7
0.6	1779	1775	6	4	99.6	99.8	99.4
0.5	1779	1775	12	4	99.3	99.8	99.1
0.4	1779	1775	17	4	99.0	99.8	98.8
mean	1779	1774	6	5	99.6	99.7	99.4

**Table 4:** The precision, recall and accuracy of the grain detection model (No. 1) and the original Faster R-CNN method (No.2) and SSD method (No.3).

No.	Manual Grain Counting	True Positive	False Positive	False Negative	Precision (%)	Recall (%)	Accuracy (%)
1	1779	1770	0	9	100.0	99.5	99.5
2	1779	1707	3	72	99.8	95.9	95.7
3	1779	1324	0	455	100.0	74.4	74.4

**Table 5:** The detection results of images with different numbers of grains.

The Number of Grains in an Image	Total Number of the Images	Manual Counting	True Positive	False Positive	False Negative	Precision (%)	Recall (%)
1–9	71	500	500	0	0	100.0	100.0
10–14	47	578	575	0	3	100.0	99.5
>14	42	701	695	0	6	100.0	99.1
Total	160	1779	1770	0	9	100.0	99.5

**Table 6:** The detection results of images taken under different lighting conditions.

Lighting Condition	Total Number of Images	Manual Counting	True Positive	False Positive	False Negative	Precision (%)	Recall (%)
Sunny	97	1024	1017	0	7	100.0	99.3
Cloudy	63	755	753	0	2	100.0	99.7
Total	160	1779	1770	0	9	100.0	99.5

**Table 7:** The detection results of grains with different dry humidity.

Grain Moisture Condition	Total Number of Images	Manual Counting	True Positive	False Positive	False Negative	Precision (%)	Recall (%)
Fresh	35	446	444	0	2	100.0	99.6
Dry	35	446	443	2	3	99.6	99.3
Total	70	892	887	2	5	99.7	99.4

## Data Availability

The data presented in this study are available on request from the corresponding author.
